# Selenium and Zinc Oxide Multinutrient Supplementation Enhanced Growth Performance in Zebra Fish by Modulating Oxidative Stress and Growth-Related Gene Expression

**DOI:** 10.3389/fbioe.2021.721717

**Published:** 2021-10-06

**Authors:** Dawit Moges Fasil, Hamida Hamdi, Amal Al-Barty, Abeer Abu Zaid, S. K. S. Parashar, Biswadeep Das

**Affiliations:** ^1^ School of Biotechnology, Kalinga Institute of Industrial Technology, KIIT Deemed to Be University, Bhubaneswar, India; ^2^ Department of Biology, College of Science, Taif University, Taif, Saudi Arabia; ^3^ Department of Biology, Alkhormah University College, Taif University, Taif, Saudi Arabia; ^4^ School of Applied Science, Kalinga Institute of Industrial Technology, KIIT Deemed to Be University, Bhubaneswar, India

**Keywords:** zebra fish, nano-supplementation, selenium, zinc oxide, growth-related genes, productivity

## Abstract

Selenium and zinc are important dietary micronutrients having antimicrobial and antioxidant roles, thereby assisting in normal development, and an enhanced immune system. Supplementation of selenium and zinc for enhancing the growth performance and reproductive capacity in fish was explored in this study. Selenium nanoparticles (SeNPs) and zinc oxide nanoparticles (ZnONPs) were synthesized by high-energy ball milling (HEBM) using a 10-h dry milling technique at a 10:1 ball-to-powder ratio (BPR) and were premixed with basal feed followed by the administration to adult zebra fish (*D. rerio*) (2 months old) for 30 days. Growth analysis revealed that zebra fish fed with SeNPs + ZnONPs (2 mg/ kg, equimolar mixture) had significantly higher length and weight than only SeNP (2 mg/ kg) or ZnONP (2 mg/ kg) groups and control zebra fish (*p* < 0.05). The average length–weight relationships were assessed by estimating the condition factor (C), which was highest in the SeNP + ZnONP group (1.96), followed by a downward trend in SeNP (C = 1.15) and ZnONP (1.11) (*p* < 0.05). Relative gene expression of growth hormone and insulin-like growth factor-1 was significantly high in the SeNP + ZnONP group compared to other groups (*p* < 0.05), which indicated that combined administration of both the nanoparticles in basal feed enhanced the growth performance of zebra fish. Intracellular ROS generation was low in the combined group, followed by control, SeNP, and ZnONP groups, indicating higher concentrations of both nanoparticles, in particular, ZnONPs induced oxidative stress. Fecundity and the development of fertilized embryos were significantly high in the SeNP + ZnONP–treated zebra fish compared to only the SeNP- or ZnONP-treated group or control (*p* < 0.05). These findings indicated that supplementation of SeNP + ZnONP in basal feed could considerably improve the growth performance and development of zebra fish which could be exploited for enhancing aquaculture production.

## Introduction

The use of nanotechnology for the enhancement of agriculture and its allied fields, such as aquaculture, has been enormous in recent time (Fasil et al., 2020). The role of nano-biotechnology for the improvement and enhancement of quality and yield of aquaculture products is enormous. Nanomaterials such as nano-micronutrients are being provided as feed supplements to fish and have been shown to improve productivity and growth performance compared to bulk feed, for example, nFe, nSe, nAg, and nZnO (Khosravi-Katuli et al., 2017). The unique benefits of using nano-micronutrients are greater bioavailability and easy absorption through the microscopic cellular membranes and organelles, thereby enhancing utilization and promoting the cellular functions, compared to bulk-sized micronutrients. Among various micronutrients, selenium is a unique trace mineral that plays an important role in maintaining the development and immune status of organisms (Ellis et al., 2004; Vicenti et al., 2018). Studies utilizing SeNPs have been conducted for aquaculture production owing to its versatile benefits, such as increased growth performance in *C. gibelio* (Zhou et al., 2009), ameliorated oxidative stress, and improved productivity in *Ctenopharyngodon idellus* (Liu et al., 2018), increased muscle composition and productivity in stress-resilient fish (Sarkar et al., 2015), improved blood biochemical profile and antioxidant status of *Cyprinus carpio* (Ashouri et al., 2015), improved growth performance, glutathione peroxidase (GSH-Px) activity, and histopathology in juvenile *Lates calcarifer* Block (Ilham and Fotedar, 2017), and in chemoprevention in commonly found fish (Allen and Gatlin, 1996; Rajendran, 2013; Swain et al., 2016). Besides, SeNPs have also been reported for improving the health and nutrition of fish, for example, it assists in maintaining the fish gill membrane integrity, enhances fish nutrition (Nastova et al., 2014), and assists in the improvement of the nutritional value of commercial fish, like tilapia (*Oreochromis niloticus*) (Moár et al., 2012; Jim et al., 2017). Importantly, Se acts as an essential as well as a toxic element having a narrow margin of tolerance in biological systems (Wang et al., 2012). Excess serum concentrations of Se can render health risks, and acute selenium toxicity can cause severe gastrointestinal and neurological symptoms, acute respiratory distress syndrome, myocardial failure, and, in very rare cases, death in living organisms (NIH, 2013). Similarly, deficiency of Se can result in various complications, including cancer, Kashin–Beck disease (a form of heart disease), fatigue, stunted growth, high cholesterol, compromised immune system function, liver impairment, pancreatic insufficiency, thyroid hormone imbalance, and sterility.

Therefore, proper dosage of Se supplementation in the basal diet is important for many organisms, including fish and crustaceans, for maintaining the proper balance of cellular metabolism (Allen and Gatlin, 1996; Rajendran, 2013; Kieliszek and Błażejak, 2016; Swain et al., 2016). Zinc (Zn) is another trace element known to have antibacterial, health-promoting, and anti-sterility effects in organisms. The most common and effective Zn-based nanoparticle are zinc oxide nanoparticles (ZnONPs) that are supplemented in a basal diet for improving the quality and health of fish (Wang, et al., 2008). ZnO toxicity studies performed in fish (Wong et al., 2010) and embryonic zebra fish (Bai et al., 2010; Bruna et al., 2014; Wehmas et al., 2015; Verma et al., 2017) revealed a dose-dependent range of toxicity and hence proposed to administer ZnONPs at moderate concentrations to reduce the toxic outcome on the growth performance (Franklin et al., 2007; Lin and Xing, 2007; Wang, et al., 2008). Most studies have assessed the fish productivity using either SeNPs or ZnONPs separately (Allen and Gatlin, 1996; Moár et al., 2012; Rajendran, 2013; Bruna et al., 2014; Wehmas et al., 2015; Swain et al., 2016; Jim et al., 2017). However, this raises the issue of increased dose administration to get the desired effects (Chris et al., 2018). Hence, it would be ideal to assess the combined efficacy of SeNPs and ZnONPs for evaluating fish growth performance and productivity under standard conditions. This study aims to throw insights into the growth performance and development of zebra fish upon administration of biologically relevant concentrations of SeNPs + ZnONPs. Because zebra fish is a freshwater teleost, having a well-defined developmental cycle; hence, assessing the efficacy of the nano-mixtures in zebra fish could be an indicator of the efficacy of the nano-mixture for aquaculture enhancement. Besides, zebra fish is considered a close relative to humans owing to 70% genome similarity in the form of orthologous genes and so can be modeled to simulate assays that can be explored further in humans. Therefore, we aim to enhance the growth performance and productivity in wild-type zebra fish using a combinatorial approach of SeNP + ZnONP supplementation in the basal feed that will have large-scale implications for enhancing aquaculture and growth of commercial fish.

## Materials and Methods

### Synthesis of SeNPs and ZnONPs

Selenium (>99.9 % trace metal) was purchased in the form of pellets < 5 mm, whereas ZnO (99.9 % trace metal) in fine powder from Sigma-Aldrich, United States. A high-energy ball milling (HEBM) technique with 300 rpm (rounds per minute) for 10 h at 28°C was utilized for nanoparticle synthesis by milling Se and ZnO separately in tungsten carbide jars (250 ml) using hardened tungsten carbide (10 mm) balls in a planetary high-energy ball mill (Retsch, PM400) at 25 Hz at the nano-lab facility of the School of Applied Sciences, KIIT Deemed to be University, Bhubaneswar, India. The BPR (ball-to-powder ratio) was maintained at 10:1 for both Se and ZnO. HEBM was employed to synthesize the NPs as it renders high purity and productivity (Verma et al., 2018).

### Characterization of SeNPs and ZnONPs

Characterization of the synthesized NPs was performed by assessing the size, size distribution, and charge to evaluate stability. TEM was used to determine the size of SeNPs and ZnONPs after pretreatment. The XRD technique was utilized to assess the size distribution using an X-ray diffractometer (X-PERT-PRO, Pan Analytical) comprising a range of angles from 15° to 75° and with CuKα radiation (λ = 0.15418 nm). A Voigt peak profile analysis was utilized to estimate the average size of the milled crystalline ZnO powders after eliminating instrumental broadening and the strain contribution. The crystalline size of NPs was determined by using Debye–Scherrer’s formula: D = Kλ / βcosѲ, where “D” designates the crystallite size; “K” is Scherrer’s constant, which shows the unity order for a normal crystal; “β” implies the full width at half maximum; and “Ѳ” is the diffraction angle. The stability of the synthesized NPs was assessed by evaluating the zeta potential of the mechanically synthesized SeNPs and ZnONPs in Milli-Q water using a Zetasizer Nano-system (Malvern Instruments, United Kingdom). The nanoparticles were then stored at various concentrations and mixed with fish feed for assessing the growth performance and development of zebra fish.

### Zebra Fish Setup and Experimental Diet Administration

All animal procedures were approved by the relevant guidelines of the Institutional Animal Ethics Committee (IAEC) of KIIT University. Adult wild-type zebra fish of 2 months old were maintained in the stand-alone aquarium setup (Aquaneering Inc, United States) at 28.0 ± 1°C, 12-h:12 h dark/light cycle, and fed a protein-rich diet (Tetra flakes powder) twice daily. A total of 500 adults (equivalent male: female fish) with an average body weight of 0.2 g and length of 23 mm were selected for this study. The fish were divided randomly into four groups depending on the type of feed provided: control (basal feed), the SeNP + basal feed group, the ZnONP + basal feed group, and the SeNP + ZnONP + basal feed group. The dosing concentrations of the nanoparticles used were 2 mg SeNPs/kg basal feed, 2 mg ZnONPs/kg basal feed, and 1 mg/kg SeNPs + 1 mg/ kg ZnONPs in basal feed. The concentrations were selected based on a concentration dependent toxicity analysis in zebra fish. The basal feed comprised ground tetramine flakes (70% protein), being administered twice a day. Fish were fed twice daily for 30 days and maintained in system water (75 gm NaHCO_3_ + 18 gm of sea salt + 8.4 gm CaSO_4_ per 1000 L). The fish were inspected daily for any mortality and were noted accordingly. The whole experiment was performed in replicates.

### Growth Performance Parameters Analysis

Zebra fish toxicity was observed by assessing the survivability (%) and any possible physical and biological alterations. The growth of the adult fish was measured in terms of weight gain and changes in body length. The total length (TL) was measured to the nearest 0.1 mm using a 10-cm ruler as the distance from the tip of the anterior-most part of the body to the tip of the caudal fin. Analytical balance with a precision of 0.01 gm was used to measure body weight (BW). The growth parameters were assessed at the beginning and the end of the experiments. Fish were anesthetized using the tricainization protocol (Westerfield, 2007; Nathan, 2018) before the experiments and then put back in normal water. The length–weight relationship was determined by linear regression analysis, and scatter diagrams of length and weight were plotted. The length–weight relationship of the experimented fish is worked out as per cube law: W = aL^b^, given by Le Cren (1951), where: “W” is weight of fish (gm), “L” is the total length (cm), “a” is the regression intercept, and “b” is the regression slope. Among the many models used to determine the well-being and condition of fish, the Fulton’s condition factor (FCF), which assumes isometric growth, is accurate for smaller-sized fish like zebra fish (Bruna et al., 2014). FCF was computed based on the equation: C= W/L^3^ * 100 (Htun-Han, 1978), where, “W” is weight in gm and “L” is length (TL) in cm.

### Intracellular ROS Analysis Using Flow Cytometry

A quantitative analysis of intracellular ROS production in the zebra fish cells was conducted using carboxy-H2DCFDA. In brief, one zebra fish/group was homogenized thoroughly for single isolation by sonication. Then, the cell supernatant was passed through a 70-micron strainer and stained with carboxy-H2DCFDA ROS indicator dye, followed by flow cytometry analysis in the BD FACS Canto II flow cytometer to determine the level of intracellular reactive oxygen species (ROS). The experiments were performed in replicates using five zebra fish/groups, and the results obtained were averaged.

### Gene Expression Analysis Using qRT-PCR

The relative expression of growth-promoting genes, growth hormone (GH), and insulin-like growth factor-1 (IGF-1) were assessed using qRT-PCR with beta-actin as the reference gene. A zebra fish from each of the four groups was grinded and homogenized thoroughly, followed by RNA extraction using the TRIzol reagent. The RNA was then converted to cDNA using a reverse transcription kit (Qiagen, United States). A SYBR Green–based qPCR reaction was performed using specific primers (Supplementary Table S1) for cDNA amplification. The thermal profile of the PCR reaction consisted of an initial denaturation of at 95°C for 2 min, followed by 30 cycles of 30 s at 95°C, 30 s at 58–62°C (varied as per primers), and 30 s at 72°C, and final extension at 72°C for 3 min. Cycle threshold (Ct) values were calculated for each gene and were compared with the reference gene beta-actin for assessing the relative expression, and ΔΔCt, and fold change (2^−ΔΔCt^) was estimated to assess the change in the gene expression among the different groups. A total of 10 zebra fish were taken from each group, and the gene expression data obtained were averaged.

### Fecundity and Development Analysis

The control zebra fish and nanoparticle-treated zebra fish of all groups were used for spawning and egg production separately after the experimental period. Zebra fish embryos were obtained by natural mating following the standard protocol. Pairs of 10 fish from all the experimental and control groups were used for mating (using 1:1 female: male ratio) and spawning. Viable eggs and healthy embryos were collected and rinsed repeatedly using the system water. The selected eggs were placed in a petri dish filled with embryo water: 5 mM NaCl, 0.17 mM KCl, 0.33 mM CaCl_2_, and 0.33 mM H_2_SO_4_. The plates were covered with transparent well cover and incubated at a temperature of 28°C at a 14/10-h light/dark photoperiod. The collected embryos were checked for survivability and development up to 5 days post-fertilization (dpf). Morphological deformities during embryonic development were recorded, as previously described (Verma et al., 2016; Verma et al., 2017), with modifications that included analysis of the backbone morphology (spinal structure), body length, optic vesicle, heart, swim bladder, yolk sac, and craniofacial morphology. The hatching rate, viability rate, and heartbeat rate were also measured using a stereomicroscope (Nikon, Japan).

### Statistical Analysis

Statistical analysis was performed using the Statistical and Data Analysis Software Package: Minitab 17 Statistical Software, (2010) and Microsoft Excel. The data obtained from the growth performance analysis, gene expression analysis, and fecundity and development analysis were reported as the mean ± SD for all experiments independently. One-way analysis of variance (ANOVA) was performed to compare the differences in the respective mean values among the groups for all the assays, followed by using the multiple comparisons test. A *p*-value of < 0.05 was considered statically significant.

## Results

### Characterization of Synthesized Se and ZnO Nanoparticles

**FIGURE 1:**
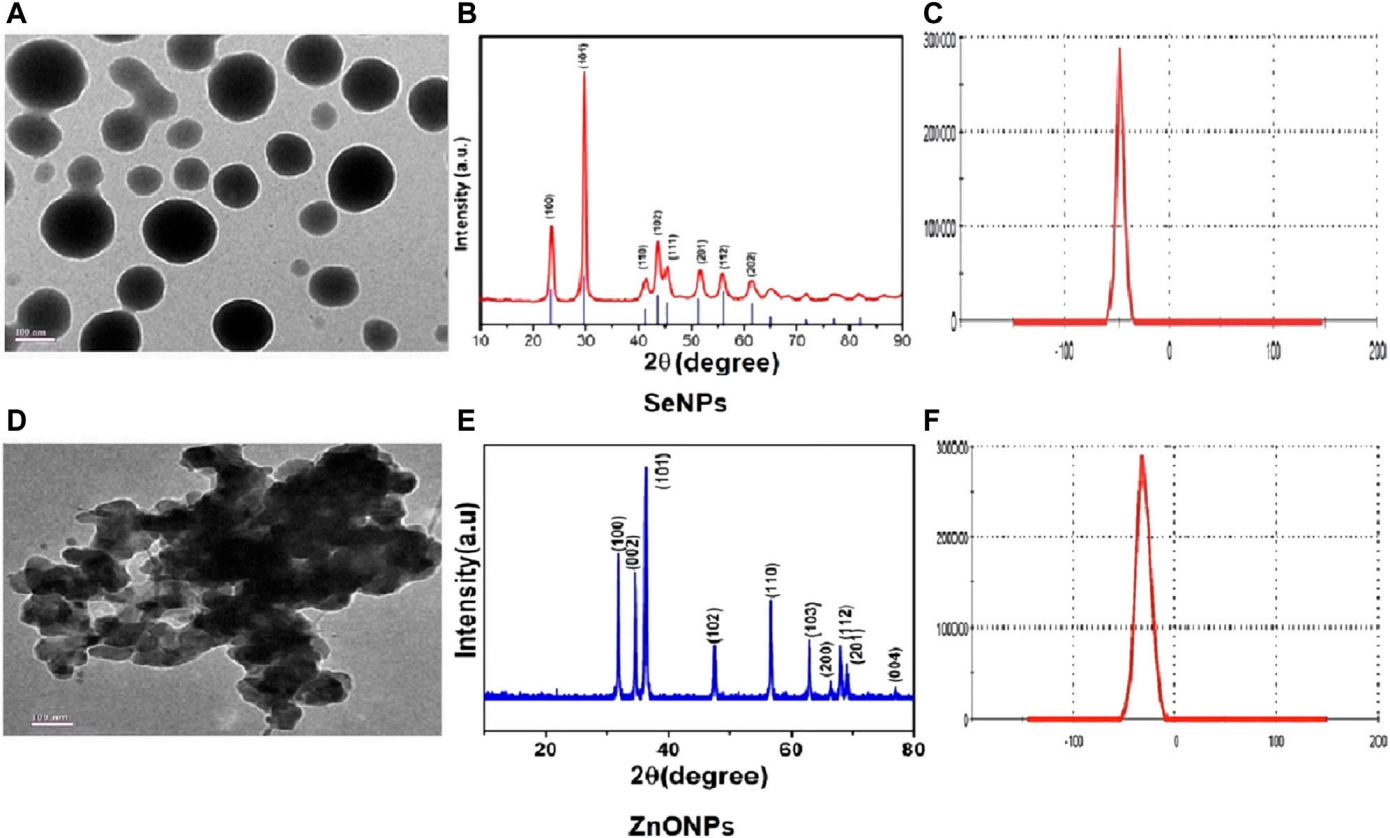
Physicochemical characterization showing the representative images of TEM **(A)**, XRD **(E)**, and zeta potential **(B)** for assessing the stability in solution for SeNPs **(C)** and ZnONPs **(D)**.

TEM analysis revealed that SeNPs and ZnONPs exhibited nearly uniform spheres having an average size 35–50 nm. Specifically, the mean size of SeNPs and ZnONPs in aqueous solution was 40 ± 12 nm and 38 ± 8 nm, respectively (Figure 1). Several studies have also reported the synthesis of uniform single crystalline nanoparticles, which further aggregated to form monodisperse spherical materials (Tanna et al., 2015; Potbhare et al., 2019), which are in line with our study. XRD patterns obtained for both nanoparticles demonstrated the peaks corresponding to crystalline t-Se (COD-9008579) at 2θ values of 23.5°, 28.6°, 40.4°, 42.7°, and 45.4° (Figure 1) corresponding to the crystal planes (100), (101), (110), (102), and (111), respectively. The average particle size of SeNPs measured by Scherrer’s equation was about 31 nm. ZnONPs peaks were located at 31.84°, 34.48°, 36.33°, 47.63°, 56.71°, 62.96°, 68.13°, and 69.18°, and the average particle size was about 42 nm. The diffraction peaks of both SeNPs and ZnONPs obtained were well-defined and were in agreement with the standard XRD patterns of SeNPs and ZnONPs (JCPDS 06–0362; JCPDS 36–1451). Zeta potential assessment of the synthesized SeNPs and ZnONPs for evaluating the stability and the aggregation effect in aqueous solution (Milli-Q water) revealed the zeta potentials as −50 ± 07 mV and −62 ± 05 mV, respectively, thereby indicating that both the NPs were stable in aqueous solution (Figure 1). The hydrodynamic size distribution was 238 ± 14 nm and 252 ± 15 nm for SeNPs and ZnONPs, respectively, at 10 h of milling time (Supplementary Figure S1).

### Survivability of Adult Zebra fish After Treatment

The survivability of zebra fish significantly varied across the groups exposed to different concentrations of SeNPs and ZnONPs. Mortality was significantly high in the ZnONP-treated group at 2 mg/ kg, followed by SeNPs at 2 mg/ kg, SeNP + ZnONP (2 mg/ kg each), and the control group. Average mortality rates of 28.90 and 44.29% were observed throughout the experimental period for SeNPs and ZnONPs, respectively. The average mortality in the combined SeNP + ZnONP group (2 mg/ kg) was 11.2%, followed by 7.3% in the control group during the experimental period (Supplementary Figure S2), which indicated that moderate concentrations of SeNPs and ZnONPs in basal feed improved the survivability rates.

### Growth Performance, Length–Weight Relationship, and Condition Factor of Zebra Fish

#### Growth Performance

The results of growth performance and feed utilization after the experimental period are shown in Table 1. Maximum growth performance was observed in the combined SeNP + ZnONP group, which was significantly higher than that in all other groups (*p* < 0.05). The females on an average were slightly longer and heavier than the males (Table 1). Moreover, the experimental groups reacted differently to different concentrations of nanoparticle exposures. In these regards, the greatest length was observed in the fish fed with the combined dose of SeNPs + ZnONPs (2 mg/ kg) compared to other groups.

**TABLE 1:** Length (L)–weight (Wt) and the condition factor (C) and sex separated for each treatment and the control group.

Group	Male			Female		
	L (mm)	Wt (mg)	C	L (mm)	Wt (mg)	C
Control	26.93	214.10	1.10	26.99	217.80	1.11
ZnONPs	24.37	237.26	1.11	25.17	247.26	1.12
SeNP	26.86	271.85	1.14	27.14	277.61	1.16
SeNPs + ZnONPs	35.42	383.25	1.93	36.71	397.26	1.98

#### Length–Weight Relationship

**FIGURE 2:**
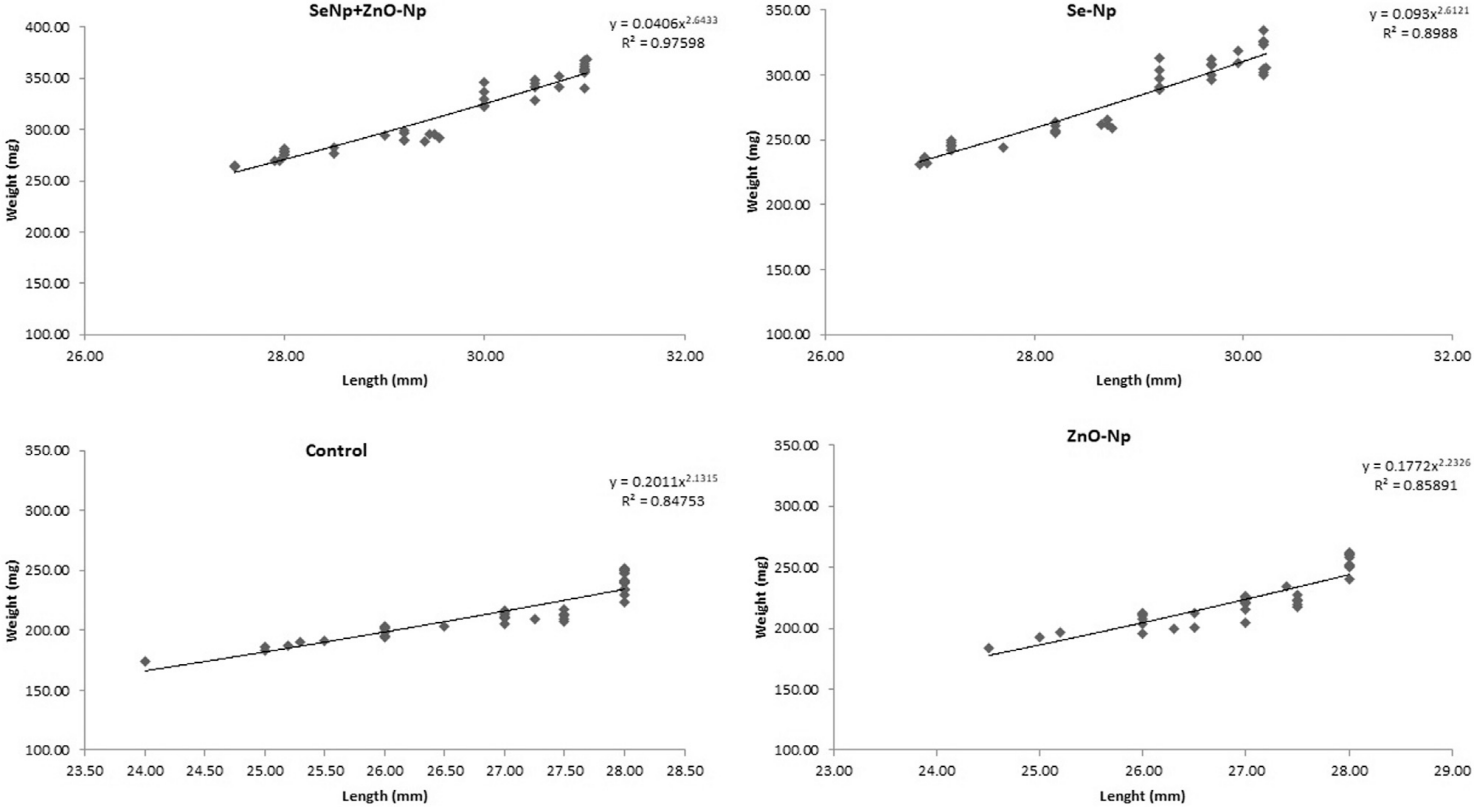
Length–weight relationship of adult zebra fish in the four groups: SeNPs + ZnONPs, SeNPs, ZnONPs, and control, showing R^2^ as the critical growth parameter value.

Length–weight statistical models were used to predict growth in terms of weight and length and to assess nutritional status as defined by the condition factor. The length–weight relationship (LWR) and the condition factor are useful biological tools that have been applied to examine the effect of any environmental changes: may be due to the feed-in use, the water chemistry, etc., on the well-being of fish under consideration (Bruna et al., 2014). We studied the growth pattern and the condition factor of zebra fish based on the types and level of concentrations of dietary NPs after being treated with different concentrations of SeNPs and ZnONPs for 4 weeks. LWR was calculated using the equation: W = aTL^b^, where W is the total weight (mg) and TL (mm), while “a” is constant and “b” is the allometric coefficient or the regression slope. The values “a” and “b” were estimated using Microsoft Excel. The average weight and length for the fish supplemented with the treatment groups, and the control group is mentioned in Tables 1, 2. SeNPs + ZnONPs treatment revealed maximum growth of zebra fish, with maximum length and weight attributes in both sexes, whereas SeNPs showed the lowest growth (Table 1). The average length (mm) and weight (mg), regression slopes (b), and the coefficient of determination (R^2^) were highest for the SeNP + ZnONP group, followed by Se-NP, ZnONPs and the control group. (Table 2; Figure 2). Generally, the females were longest and heaviest than the corresponding males, and treatment with dietary nanoparticles showed better performance than that of the control group (Tables 1, 2).

**TABLE 2:** Growth parameters like average length (L) (mm) and weight (Wt) (mg) and the condition factor (FCF) for each treatment. Values (C, L, and Wt) are mean ± standard deviation.

Parameter/attribute	Control	SeNP + ZnONP	ZnONP	SeNP
B	2.23	3.24	2.31	2.52
R^2^	0.84	0.97	0.85	0.88
C	1.10 ± 0.01	1.96 ± 0.11	1.11 ± 0.03	1.15 ± 0.16
Av. L.(mm)	26.96 ± 0.11	36.17 ± 0.44	24.87 ± 0.37	26.8 ± 0.31
Av. Wt. (mg)	215.95 ± 6.02	393.23 ± 11.14	242.26 ± 13.98	273.73 ± 14.35

### Condition Factor/Well-Being of Zebra Fish

The condition factor, C, was highest in the SeNP + ZnONP group (1.96), followed by a downward trend in SeNP (K = 1.15) and ZnONP (1.11) (*p* < 0.05). SGR and WGR displayed significant quadratic responses to graded levels of dietary SeNPs and ZnONPs based on polynomial orthogonal contrast (*p* < 0.05) (Figure 2). These findings indicated that combined supplementation with SeNPs + ZnONPs revealed better growth performance than other treatments including the control group (Table 2.). The female fish exhibited slightly better growth conditions than the males.

### Intracellular ROS Analysis

**FIGURE 3:**
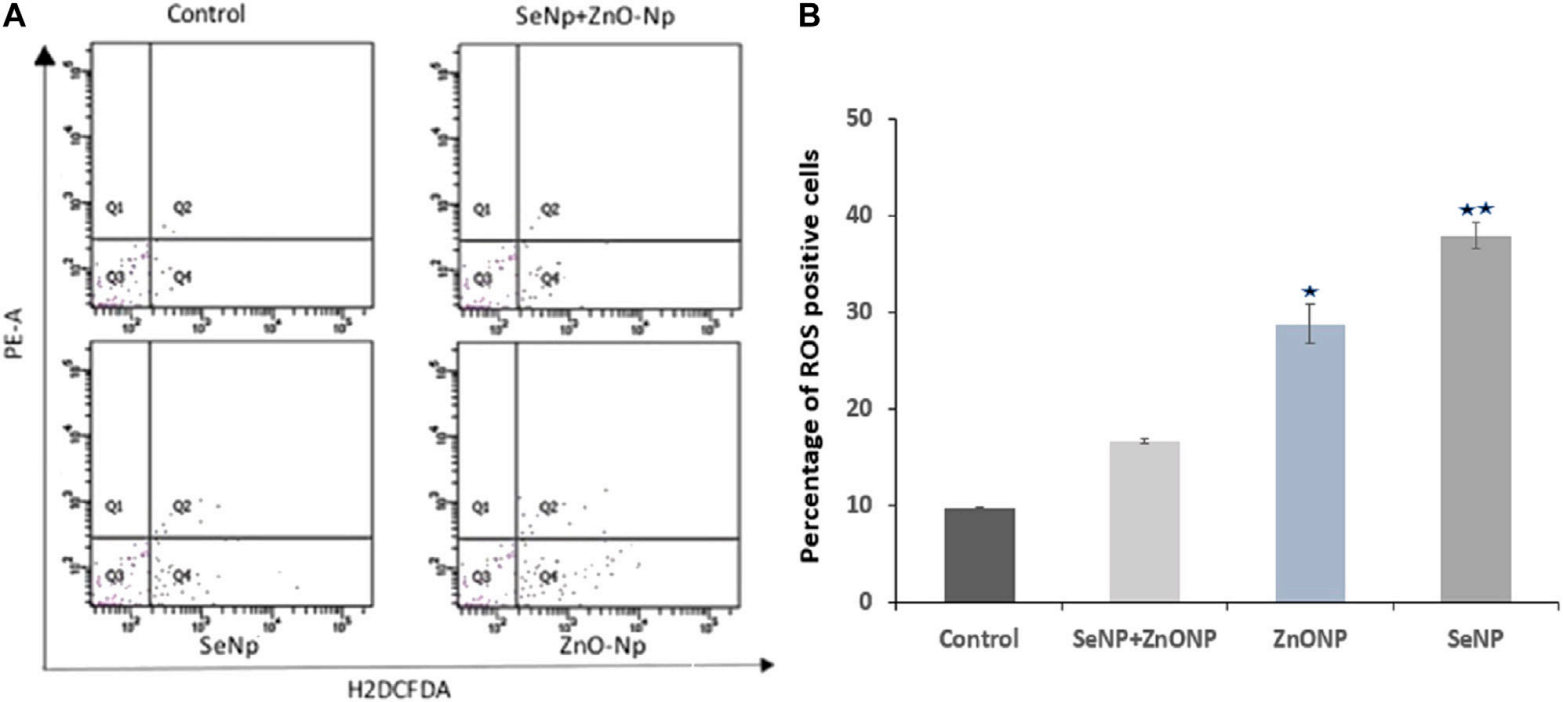
Intracellular ROS analysis in adult zebra fish using flow cytometry: **(A)** Q4 represents the intracellular ROS level measured by H2DCFDA fluorescence dye.**(B)** Bar graph showing the percentage of ROS + cells (Q4 quadrant of the flow cytometric graph) in the four different groups. Significantly low mean value ± SD of ROS + cells were found (*p* < 0.05) in both ZnONP and SeNP groups compared to control and ZnONP + SeNP groups. *p < 0.05 denote significant variation from control embryos as obtained by ANOVA analysis.

To explore the effect of SeNPs and ZnONPs on ROS production in zebra fish, intracellular ROS production was quantified using the ROS detection reagent (H2DCFDA). Intracellular ROS flow cytometry analysis revealed no/minimum variations in the mean DCFDA fluorescence in the zebra fish treated with different combined SeNPs + ZnONPs (2 mg/ kg each) concentrations and control, whereas the mean DCFDA fluorescence was significantly high in the zebra fish treated with only ZnONPs (2 mg/ kg), followed by that treated with SeNPs (2 mg/ kg) (Figure 3). This finding showed that combined administration of SeNPs + ZnONPs generated less ROS than a single administration of either nanoparticle.

### Gene Expression Analysis Upon IOP Exposure in Zebra Fish Embryos

**FIGURE 4:**
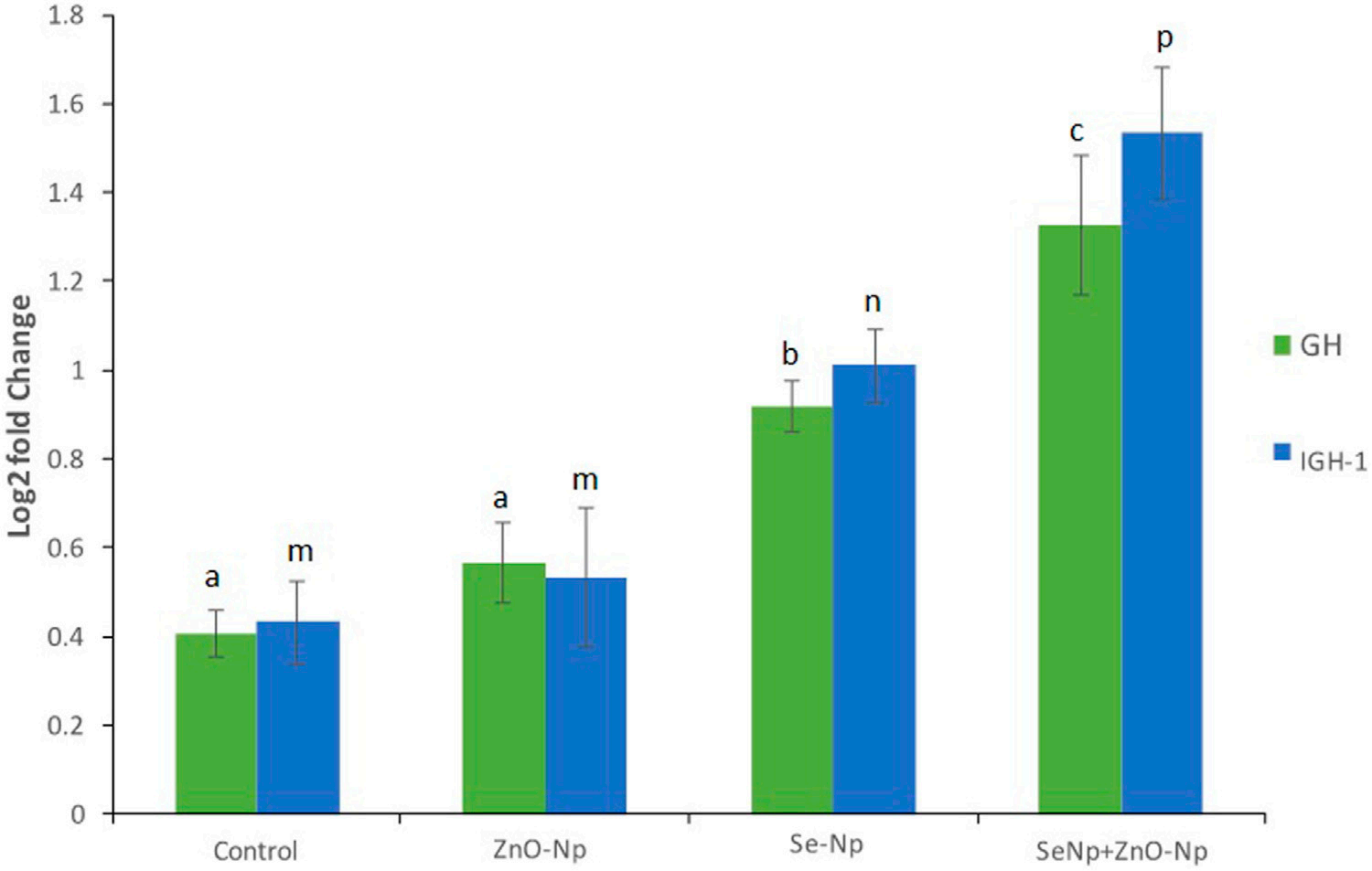
qRT-PCR bar graphs showing the log2 fold change in the growth-related genes growth hormone (GH), and insulin-like growth factor-1 (IGF-1) expression in the different groups, showing significantly upregulation in the SeNPs + ZnONPs group compared to all groups (*p* < 0.001).

Relative expression of GH and IGF-1 assessed using qRT-PCR was significantly upregulated in the significantly upregulated in the SeNPs + ZnONPs group compared to all groups, followed by moderate upregulation in the SeNPs groups. Control and ZnONPs groups showed similar expression of both the genes (Figure 4). Such findings indicated that combined administration of both SeNPs and ZnONPs in fish feed enhances the growth of the zebra fish through enhancing the expression of the growth-promoting genes, which are essential for the proper growth and development of zebra fish.

### Fecundity and Development Analysis

**FIGURE 5:**
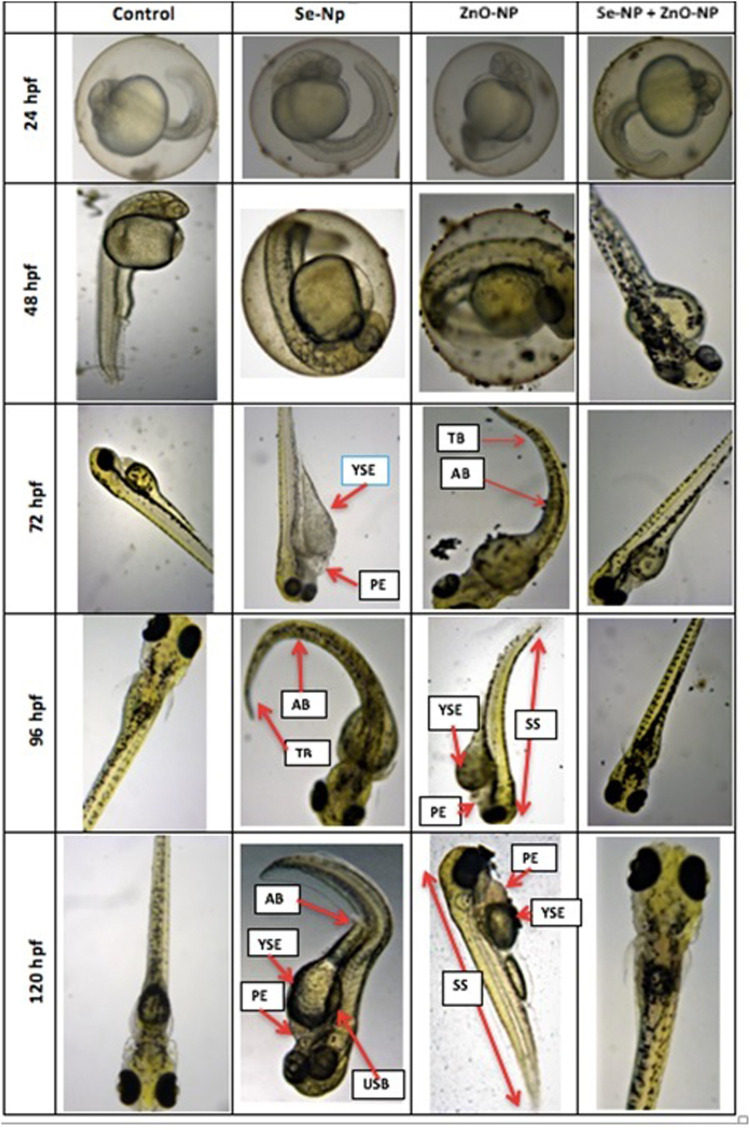
Morphological analysis of zebra fish embryos obtained from the four different groups (Control, SeNPs, ZnONPs, and SeNPs + ZnONPs). The structural deformities are shown in arrows: SC: spinal curvature, TM: tail malformation, PE: pericardial edema, YSE: yolk sac edema, USB: uninflated swim bladder, AX: axis (scoliosis), TB: tail bending, and SS: small size (dwarf).

The embryo density averaged at 72 ± 8, 105 ± 5, 130 ± 12, and 102 ± 10 live and fertilized embryos in ZnONPs, SeNPs, SeNPs + ZnONPs, and control groups, respectively, after repeated mating of the males and females of the specific group, which revealed maximum fecundity in the zebra fish treated with both SeNPs + ZnONPs. During further embryonic development, morphological deformities varied across the zebra fish embryos, in particular considerable morphological deformities, prolonged hatching time, and less survivability were observed in the ZnONPs group, followed by the SeNPs group. Control and SeNP + ZnONP groups developed normally. The survival rate and hatching rate were significantly low in the ZnONPs, followed by SeNP-treated groups, than the control and SeNP + ZnONP group (*p* < 0.05), (Figure 5). This observation could be attributed to the synergistic effect of SeNPs + ZnONPs when administered together to the parental fish, compared to the single administration of either nanoparticle. Distinct developmental deformities were observed in the zebra fish larvae of different ages. At 72 hpf, embryos of the ZnONP group and less frequently the SeNPs group showed mild to severe spinal curvature (SC), yolk sac edema (YSE), and tail malformations (TM). SC, TM, and YSE were significantly high in the ZnONP groups in comparison to other groups (Figure 5). The body length of zebra fish larvae significantly varied from 2.78 ± 0.043 mm, 2.08 ± 0 mm, 3.52 ± 0.089 mm, to 3.22 ± 0.089 mm in the SeNP, ZnONP, SeNP + ZnONP, and control groups (*p* < 0.05).

## Discussion

**FIGURE 6:**
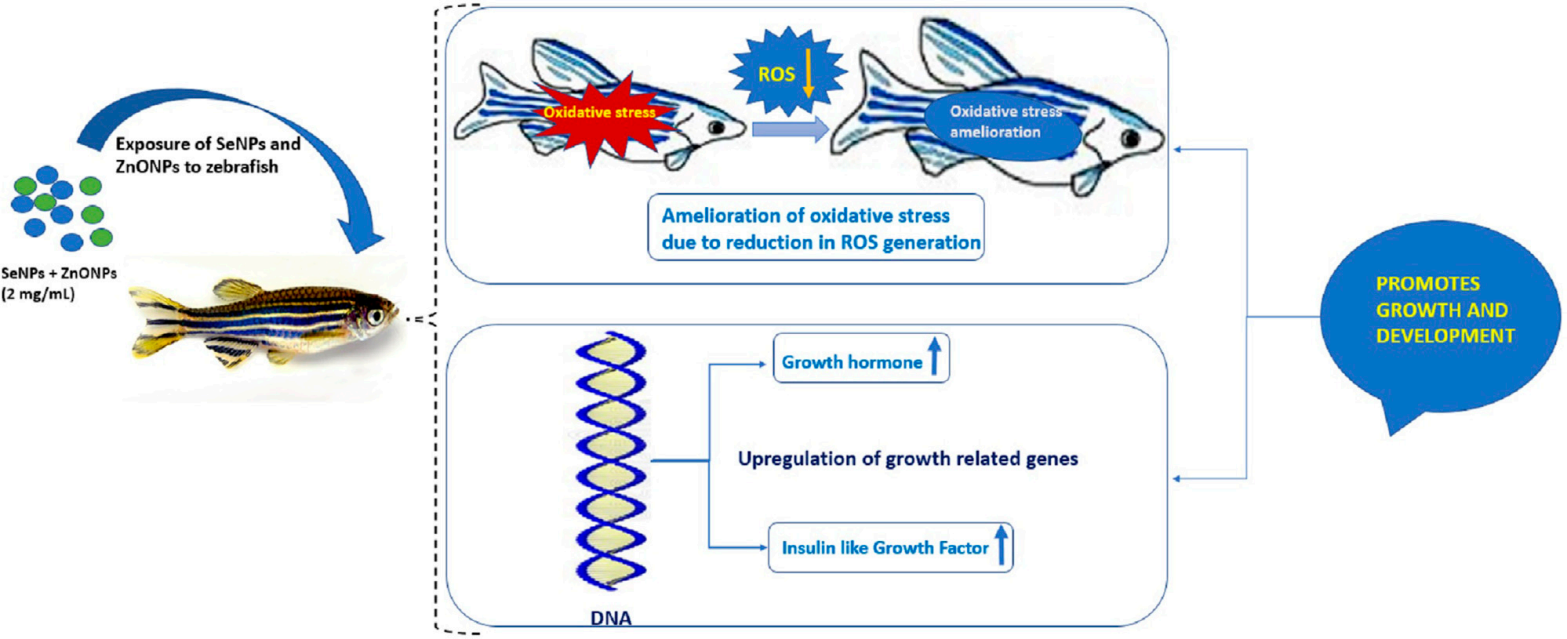
Mechanistic diagram delineating the effect of combined use of SeNP and ZnONPs on zebra fish exhibiting higher growth performance due to reduction of oxidative stress (OS) and ROS and upregulation of growth-regulated genes.

Aquaculture enhancement through the utilization of unique components, such as fermented materials, nanoparticles, trace elements, and many others, is currently been pursued to improve fish production to address the needs of malnutrition and better nutrition to the community (Fasil et al., 2020). In this regard, nanoparticles of trace elements could be utilized for the overall growth and development of fish through appropriate supplementation in fish feed, which is a handy and natural way of delivery to the fish. The current study showed that the combined administration of SeNPs + ZnONPs could lead to enhanced growth performance and improved development in zebra fish, whereas single administration of either nanoparticle results in subtle growth, increased oxidative stress, and reduced growth-related gene expression. Adult zebra fish growth performance was significantly enhanced by the administration of SeNPs and ZnONPs as revealed by the increased growth analysis parameters, such as Fulton’s condition factor, which was observed to be highest in the combined group and lowest in the control group. This model of growth analysis has been accepted by worldwide research for assessing growth performance in small fish, like zebra fish (Bruna et al., 2014). Several research studies have confirmed the varied roles of Se and ZnONPs separately. Studies have reported that SeNP supplementation in fish feed yielded better performance and significantly improved the growth performance and immune status of fish (Ashouri et al., 2015; Khan et al., 2016), which was in line with our study. In addition, SeNP supplementation was observed to improve the productivity and antioxidant status of crucian carp (Zhou et al., 2009; Saffari et al., 2016). Similar growth performance observation was observed in teleosts; recently, a study reported that Se supplementation resulted in better growth of the fish (Bai et al., 2019). Some researchers reported that Se administered even in relatively large doses had minor adverse effects on the growth performance of African catfish (*Clarias gariepinus*) (3.67 mg Se/ Kg; Abdel-Tawwab et al., 2007); however, contradictory studies reported better growth performance for some other related species, such as lower dose worked better for loach (0.48–0.50 mg Se/ kg; Hao et al., 2014) and grouper (0.77 mg Se/ kg; Lin and Shiau, 2005). Such variation in effects of Se concentrations can be attributed to diverse levels of Se requirement or the level of toxicity related to species fish and age of fish, the type and chemical form of Se, and other factors like dietary factors (Jim et al., 2017; Liu, et al., 2010, Hao et al., 2014). Similarly, an earlier study done on ZnONPs exposed to several fish species showed increased respiratory burst activity in tilapia, suggesting ZnONP ability to induce oxidative stress (Kaya et al., 2015). In this study, a single administration of ZnONP (2 mg/ Kg basal feed) showed greater mortality rates and increased oxidative stress, in addition to low fecundity and growth-related gene expression, suggesting that only ZnO supplementation retards the growth performance and productivity of zebra fish (Table 2.). One reason for this could be the ion-shedding property of ZnO as they are partially soluble in neutral solutions, which can cause the formation of reactive oxygen species (ROS). On the contrary, studies have shown that ZnO in stabilized nano-form has a higher bioavailability and could be more readily absorbed in the gastrointestinal tract of the African catfish (*Clarias garipinus*) fingerlings than its bulk. In another experiment involving Indian major carp, *L. rohita*, the effect of the combination of ZnO and SeNPs on growth, immunological, and serum enzymatic profiles was found to yield great benefits (Ibrahim, 2020). Hence, it is imperative to conduct combined administration of such nanoparticles in fish feed to assess the levels of toxicity, growth ability, productivity, and fecundity, which was performed in this study, which found synergistic effects of combined use of both nanoparticles in zebra fish. Because both ZnO and Se have antioxidant properties, supplementation of these two nutrients in combination will stimulate each other’s effect and will also boost the overall growth performance and health of zebra fish (Figure 6). To further unravel the synergistic effects of both nanoparticles at the molecular level, qPCR was performed on growth hormone (GH) and insulin-like growth factor-1 (IGF-1) that are important markers of growth in fish in nutrigenomics studies (Asaduzzaman et al., 2017; Hoseinifar et al., 2018; Zemheri-Navruz et al., 2020). GH plays a critical role in regulating several physiological processes, such as maintaining osmotic balance, carbohydrate, protein, and lipid metabolisms, soft tissue growth, immune function, and reproduction. IGF-I enhances the growth, development, and differentiation of fish and other aquatic species (Duan, 1998; Reinecke, 2010). Enhanced expression of both GH and IGF-1 in the combined SeNP + ZnONP group, whereas low expression of both genes in the single-dose group suggested that both SeNP and ZnONP conferred synergistic effects on the growth-related gene expression in zebra fish, which eventually promoted growth performance and productivity.

Developmental toxicity analysis of the treated and control groups showed certain manifestations such as high mortality, delayed hatching, pericardial edema, yolk sac edema, spinal curvature, tail bending, uninflated swim bladder, and diminished body size in the zebra fish embryos of the single-dose ZnONP group, followed by the SeNP group. This was in agreement with Choi et al. (2016) and Hua et al. (2014), who reported reduced growth and developmental abnormalities in fish using single doses of dietary nanoparticles. Besides, the combined administration of SeNPs + ZnONPs in the parents produced embryos that showed proper development and enhanced the hatching rate compared to single-dose administration of the corresponding nanoparticles. Such effects could be attributed to the synergic effects of the combined SeNPs + ZnONPs. The mortality and severity of malformations exposed to SeNPs and ZnONP increased in single administration for continuous periods. These findings suggested that the acute toxicological effects of Se and ZnONPs on zebra fish were dose- and time-dependent. A similar discussion was observed in the work of Wehmas et al. (2015), wherein the bulk counterparts were observed to be more toxic than the nanoparticles. This might be attributed to the different surface charges and surface effects, besides the increased size that can cause problems in membrane permeability (Ispas et al., 2009; Mironava et al., 2010). ZnONP appeared to be more toxic and imposed severe adverse effects on the development of the zebra fish embryos. Many pieces of research performed on the toxicity of ZnO reported that ZnONPs are toxic to zebra fish in a dose-dependent manner (Zhu, et al., 2008; Zhu et al., 2009; Zhao et al., 2013). Zhu et al. (2009) demonstrated that ZnONPs induce a concentration-dependent decrease in hatching rates. Pericardial edema and malformations were observed in ZnONP-exposed embryos (Zhu et al., 2008). Bai et al. (2010) found that exposure to nano-ZnO could decrease the hatching rates in zebra fish embryos and result in tail malformation. Liu et al. (2014) demonstrated that the toxicity effect of ZnONP confers shortening of the body length and reduction of hatching rates. The precise mechanism of higher toxicity of ZnONPs concerning SeNPs still needs to be addressed, although the hypothesis of such increased toxicity of ZnONPs could be attributed to the ion-shedding ability of ZnO because of partial solubility in neutral solutions (Liu et al., 2016), and aggregation of particles and interaction of Zn^+2^ with other ions within the culturing medium, producing reactive species that can cause tissue and organ damage (Franklin et al., 2007; Lin and Xing, 2007).

Multinutrient supplementation is currently an approach similar to multivitamin supplementation for improving the immune status and health of an organism. Selenium and zinc have antioxidant properties and are essential for the overall development, growth performance, immune status, and reproduction in organisms. Combined supplementation of both the elements at a nanoscale confers advantages such as easy absorption by the cells because of their small size, greater benefits, and lesser side effects compared to single dose owing to the high reactive potential in a free state. Our study indicated that combined SeNP and ZnONP in the basal feed of zebra fish could considerably improve the growth performance and development of zebra fish, which were also characterized by reduced levels of intracellular ROS production, increased levels of growth-related gene expression, and higher productivity, which could be exploited for enhancing aquaculture production in various types of fishes.

## Conclusion

Combined supplementation of SeNPs and ZnONPs improved the growth performance and productivity of zebra fish compared to a single administration of either nanoparticle at similar concentrations. This is because of the synergetic effects of both the nanoparticles at the molecular level by modulating the expression of growth-related genes and mitigating the production of reactive oxygen species. Because both Se and Zn have key roles in the physiology and development of an organism; hence, combined supplementation produced the best growth performance. Industries related to feed and feed supplementations can first rate the combination of Se and ZnO nanoparticles at optimum levels as substantial input for commercial fish growth enhancers. Since such studies on utilizing various combinations of such microelements are scarce, its application should be considered and explored at a higher and commercial level for addressing the challenge of the malnourished global situation.

## Data Availability

The original contributions presented in the study are included in the article/Supplementary Material; further inquiries can be directed to the corresponding authors.

## References

[B1] Abdel-Tawwab M. Mousa M. A. A. Abbass F. E. (2007). Growth Performance and Physiological Response of African Catfish, *Clarias gariepinus* (B.) Fed Organic Sselenium Prior to the Exposure to Environmental Copper Toxicity. Aquaculture 272, 335–345. 10.1016/j.aquaculture.2007.09.004

[B2] Asaduzzaman M. Ikeda D. Abol-Munafi A. B. Bulbul M. Ali M. E. Kinoshita S. (2017). Dietary Supplementation of Inosine Monophosphate Promotes Cellular Growth of Muscle and Upregulates Growth-Related Gene Expression in Nile tilapia *Oreochromis niloticus* . Aquaculture 468, 297–306. 10.1016/j.aquaculture.2016.10.033

[B3] Ashouri S. Keyvanshokooh S. Salati A. P. Johari S. A. Pasha-Zanoosi H. (2015). Effects of Different Levels of Dietary Selenium Nanoparticles on Growth Performance, Muscle Composition, Blood Biochemical Profiles and Antioxidant Status of Common Carp (*Cyprinus carpio*). Aquaculture 446, 25–29. 10.1016/j.aquaculture.2015.04.021

[B4] Bai W. Zhang Z. Tian W. He X. Ma Y. Zhao Y. (2010). Toxicity of Zinc Oxide Nanoparticles to Zebrafish Embryo: a Physicochemical Study of Toxicity Mechanism. J. Nanopart Res. 12, 1645–1654. 10.1007/s11051-009-9740-9

[B5] Bai Z. Ren T. Han Y. Hu Y. Schohel M. R. Jiang Z. (2019). Effect of Dietary Bio-Fermented Selenium on Growth Performance, Nonspecific Immune Enzyme, Proximate Composition and Bioaccumulation of Zebrafish (*Danio rerio*). Aquacult. Rep. 13, 100180. 10.1016/j.aqrep.2019.100180

[B6] Brun N. R. Lenz M. Wehrli B. Fent K. (2014). Comparative Effects of Zinc Oxide Nanoparticles and Dissolved Zinc on Zebrafish Embryos and Eleuthero-Embryos: Importance of Zinc Ions. Sci. Total Environ. 476-477, 657–666. 10.1016/j.scitotenv.2014.01.053 24508854

[B7] Choi J. S. Kim R.-O. Yoon S. Kim W.-K. (2016). Developmental Toxicity of Zinc Oxide Nanoparticles to Zebrafish (*Danio rerio*): A Transcriptomic Analysis. PLoS ONE 11 (8), e0160763. 10.1371/journal.pone.0160763 27504894 PMC4978389

[B8] Chris U. O. Singh N. B. Agarwal A. (2018). Nanoparticles as Feed Supplement on Growth Behaviour of Cultured Catfish ( *Clarias gariepinus* ) Fingerlings. Mater. Today Proc. 5, 9076–9081. 10.1016/j.matpr.2017.10.023

[B9] Cren E. D. L. (1951). The Length-Weight Relationship and Seasonal Cycle in Gonad Weight and Condition in the Perch (*Perca fluviatilis*). J. Anim. Ecol. 20, 201–219. 10.2307/1540

[B10] Davis D. A. Gatlin D. M. (1996). Dietary Mineral Requirements of Fish and Marine Crustaceans. Rev. Fish. Sci. 4, 75–99. 10.1080/10641269609388579

[B12] Duan C. (1998). Nutritional and Developmental Regulation of Insulin-like Growth Factors in Fish. J. Nutr. 128, 306S–314S. 10.1093/jn/128.2.306S 9478013

[B13] Ellis D. Sors T. G. Brunk D. G. Albrecht C. Orser C. Lahner B. (2004). Production of Se-Methyl Selenocysteine in Transgenic Plants Expressing Selenocysteine Methyl Transferase. BMC Plant Biol. 4, 1. 10.1186/1471-2229-4-1 15005814 PMC343276

[B14] FAO (2005). Fisheries Topics: Technology. *Biotechnology.* Text by Devein Bartley and Rohana Subasinghe. Rome: FAO Fisheries and Aquaculture Department (online). Available at: http://www.fao.org/fishery/topic/12316/en (Accessed May 27, 2005).

[B11] Fasil D. M. Patel P. Parashar S. K. S. Das B. (2020). Mechanistic Insights into Diverse Nano-Based Strategies for Aquaculture Enhancement: A Holistic Review. Aquaculture 519, 734770. 10.1016/j.aquaculture.2019.734770

[B15] Franklin N. M. Rogers N. J. Apte S. C. Batley G. E. Gadd G. E. Casey P. S. (2007). Comparative Toxicity of Nanoparticulate ZnO, Bulk ZnO, and ZnCl2to a Freshwater Microalga (Pseudokirchneriella Subcapitata): The Importance of Particle Solubility. Environ. Sci. Technol. 41, 8484–8490. 10.1021/es071445r 18200883

[B16] Hao X. Ling Q. Hong F. (2014). Effects of Dietary Selenium on the Pathological Changes and Oxidative Stress in Loach (*Paramisgurnus dabryanus*). Fish. Physiol. Biochem. 40, 1313–1323. 10.1007/s10695-014-9926-7 24633928

[B17] Hoseinifar S. H. Sun Y.-Z. Wang A. Zhou Z. (2018). Probiotics as Means of Diseases Control in Aquaculture, a Review of Current Knowledge and Future Perspectives. Front. Microbiol. 9, 2429. 10.3389/fmicb.2018.02429 30369918 PMC6194580

[B18] Htun-Han M. (1978). The Reproductive Biology of the Dab *Limanda limanda* (L.) in the North Sea: Gonosomatic index, Hepatosomatic index and Condition Factor. J. Fish. Biol. 13, 369–378. 10.1111/j.1095-8649.1978.tb03445.x

[B19] Hua J. Vijver M. G. Richardson M. K. Ahmad F. Peijnenburg W. J. G. M. (2014). Particle-specific Toxic Effects of Differently Shaped Zinc Oxide Nanoparticles to Zebrafish Embryos (*Danio rerio*). Environ. Toxicol. Chem. 33, 2859–2868. 10.1002/etc.2758 25244315

[B20] Ibrahim A. T. A. (2020). Toxicological Impact of green Synthesized Silver Nanoparticles and Protective Role of Different Selenium Type on *Oreochromis niloticus*: Hematological and Biochemical Response. J. Trace Elem. Med. Biol. 61, 126507. 10.1016/j.jtemb.2020.126507 32278284

[B21] Ilham I. Fotedar R. (2017). Growth, Enzymatic Glutathione Peroxidase Activity and Biochemical Status of Juvenile Barramundi (*Lates calcarifer*) Fed Dietary Fermented Soybean Meal and Organic Selenium. Fish. Physiol. Biochem. 43, 775–790. 10.1007/s10695-016-0331-2 28028742

[B22] Ispas C. Andreescu D. Patel A. Goia D. V. Andreescu S. Wallace K. N. (2009). Toxicity and Developmental Defects of Different Sizes and Shape Nickel Nanoparticles in Zebrafish. Environ. Sci. Technol. 43, 6349–6356. 10.1021/es9010543 19746736 PMC2744893

[B23] Jim F. Garamumhango P. Musara C. (2017). Comparative Analysis of Nutritional Value ofOreochromis niloticus(Linnaeus), Nile Tilapia, Meat from Three Different Ecosystems. J. Food Qual. 2017, 1–8. 10.1155/2017/6714347

[B24] Kaya H. Aydın F. Gürkan M. Yılmaz S. Ates M. Demir V. (2015). Effects of Zinc Oxide Nanoparticles on Bioaccumulation and Oxidative Stress in Different Organs of tilapia (*Oreochromis niloticus*). Environ. Toxicol. Pharmacol. 40, 936–947. 10.1016/j.etap.2015.10.001 26513690

[B25] Khan K. U. Zuberi A. Fernandes J. B. K. Ullah I. Sarwar H. (2017). An Overview of the Ongoing Insights in Selenium Research and its Role in Fish Nutrition and Fish Health. Fish. Physiol. Biochem. 43, 1689–1705. 10.1007/s10695-017-0402-z 28712005

[B26] Khan K. U. Zuberi A. Nazir S. Fernandes J. B. K. Jamil Z. Sarwar H. (2016). Effects of Dietary Selenium Nanoparticles on Physiological Andbiochemical Aspects of Juvenile Tor Putitora. Turk J. Zool 40, 704–712. 10.3906/zoo-1510-5

[B27] Khosravi-Katuli K. Prato E. Lofrano G. Guida M. Vale G. Libralato G. (2017). Effects of Nanoparticles in Species of Aquaculture Interest. Environ. Sci. Pollut. Res. 24, 17326–17346. 10.1007/s11356-017-9360-3 28597390

[B28] Kieliszek M. Błażejak S. (2016). Current Knowledge on the Importance of Selenium in Food for Living Organisms: A Review. Molecules 21, 609. 10.3390/molecules21050609 PMC6274134 27171069

[B29] Lin D. Xing B. (2007). Phytotoxicity of Nanoparticles: Inhibition of Seed Germination and Root Growth. Environ. Pollut. 150, 243–250. 10.1016/j.envpol.2007.01.016 17374428

[B30] Lin Y.-H. Shiau S.-Y. (2005). Dietary Selenium Requirements of Juvenile Grouper, Epinephelus Malabaricus. Aquaculture 250, 356–363. 10.1016/j.aquaculture.2005.03.022

[B31] Liu J. Fan D. Wang L. Shi L. Ding J. Chen Y. (2014). Effects of ZnO, CuO, Au, and TiO2 Nanoparticles on Daphnia magna and Early Life Stages of Zebrafish (*Danio rerio*). Environ. Prot. Eng. 40, 140–149. 10.37190/epe140111

[B32] Liu J. Feng X. Wei L. Chen L. Song B. Shao L. (2016). The Toxicology of Ion-Shedding Zinc Oxide Nanoparticles. Crit. Rev. Toxicol. 46, 348–384. 10.3109/10408444.2015.1137864 26963861

[B33] Liu K. Wang X. J. Ai Q. Mai K. Zhang W. (2010). Dietary Selenium Requirement for Juvenile Cobia, *Rachycentron canadum* L. Rachycentron Canadum L. Aquac. Res. 41, no. 10.1111/j.1365-2109.2010.02562.x

[B34] Liu L. W. Liang X.-F. Li J. Fang J. G. Yuan X. C. Li J. (2018). Effects of Dietary Selenium on Growth Performance and Oxidative Stress in Juvenile Grass carpCtenopharyngodon Idellus. Aquacult Nutr. 24, 1296–1303. 10.1111/anu.12667

[B35] Minitab 17 Statistical Software (2010). Computer Software. State College, PA: Minitab, Inc. Available at: www.minitab.com .

[B36] Mironava T. Hadjiargyrou M. Simon M. Jurukovski V. Rafailovich M. H. (2010). Gold Nanoparticles Cellular Toxicity and Recovery: Effect of Size, Concentration and Exposure Time. Nanotoxicology 4, 120–137. 10.3109/17435390903471463 20795906

[B37] Moár T. Biró J. Balogh K. Mézes M. Hancz C. (2012). Improving the Nutritional Value of Nile Tilapia Fillet by Dietary Selenium Supplementation. The Israeli J. Aquaculture-Bamidgeh 64 (744), 6. 10.46989/001c.20641

[B38] Nastova R. Gjorgovska N. Kostov V. (2014). Selenium Supplementation in Fish Nutrition. AgroLife Scientific J. 3 (1). Available at: http://agrolifejournal.usamv.ro/index.php/scientific-papers/14-vol-3/151-selenium-supplementation-in-fish-nutrition .

[B39] Nathan D. (2018). General Methods for Zebrafish Care. ZFIN Protocol WiKi. ZFIN Database Team. Modified by Anne Eagle on Feb 02, 2018.

[B40] NIH (2013). Selenium: Fact Sheet for Health Professionals. USA: National Institutes of Health Office of Dietary Supplements. U.S. Department of Health and Human Service.

[B41] Potbhare A. K. Chaudhary R. G. Chouke P. B. Yerpude S. Mondal A. Sonkusare V. N. (2019). Phytosynthesis of Nearly Monodisperse CuO Nanospheres Using Phyllanthus Reticulatus/Conyza Bonariensis and its Antioxidant/antibacterial Assays. Mater. Sci. Eng. C. 99, 783–793. 10.1016/j.msec.2019.02.010 30889753

[B42] Rajendran D. (2013). Application of Nano Minerals in Animal Production System. Res. J. Biotech. 8, 1–3. https://www.researchgate.net/publication/236248725.

[B43] Rayman M. P. (2012). Selenium and Human Health. The Lancet 379 (9822), 1256–1268. 10.1016/S0140-6736(11)61452-9 22381456

[B44] Reinecke M. (2010). Influences of the Environment on the Endocrine and Paracrine Fish Growth Hormone-insulin-like Growth Factor-I System. J. Fish. Biol. 76, 1233–1254. 10.1111/j.1095-8649.2010.02605.x 20537012

[B45] Saffari S. Keyvanshokooh S. Zakeri M. Johari S. A. Pasha-Zanoosi H. (2016). Effects of Different Dietary Selenium Sources (Sodium Selenite, Selenomethionine and Nanoselenium) on Growth Performance, Muscle Composition, Blood Enzymes and Antioxidant Status of Common Carp (*Cyprinus carpio*). Aquacult Nutr. 23, 611–617. 10.1111/anu.12428

[B46] Sarkar B. Bhattacharjee S. Daware A. Tribedi P. Krishnani K. K. Minhas P. S. (2015). Selenium Nanoparticles for Stress-Resilient Fish and Livestock. Nanoscale Res. Lett. 10, 371. 10.1186/s11671-015-1073-2 26400834 PMC4580674

[B47] Swain P. S. Rao S. B. N. Rajendran D. Dominic G. Selvaraju S. (2016). Nano Zinc, an Alternative to Conventional Zinc as Animal Feed Supplement: A Review. Anim. Nutr. 2, 134–141. 10.1016/j.aninu.2016.06.003 29767083 PMC5941028

[B48] Tanna J. A. Chaudhary R. G. Juneja H. D. Gandhare N. V. Rai A. R. (2015). Histidine-Capped ZnO Nanoparticles: An Efficient Synthesis, Spectral Characterization and Effective Antibacterial Activity. BioNanoSci. 5 (3), 123–134. 10.1007/s12668-015-0170-0

[B49] Verma S. K. Jha E. Panda P. K. Mukherjee M. Thirumurugan A. Makkar H. (2018). Mechanistic Insight into ROS and Neutral Lipid Alteration Induced Toxicity in the Human Model with Fins (*Danio rerio*) by Industrially Synthesized Titanium Dioxide Nanoparticles. Toxicol. Res. 7, 244–257. 10.1039/C7TX00300E PMC6061716 30090579

[B50] Verma S. K. Mishra A. K. Suar M. Parashar S. K. S. (2017). *In Vivo* assessment of Impact of Titanium Oxide Nanoparticle on Zebrafish Embryo. AIP Conf. Proc. 1832, 040030. 10.1063/1.4980232

[B51] Verma S. K. Panda P. K. Jha E. Suar M. Parashar S. K. S. (2017). Altered Physiochemical Properties in Industrially Synthesized ZnO Nanoparticles Regulate Oxidative Stress; Induce *In Vivo* Cytotoxicity in Embryonic Zebrafish by Apoptosis. Sci. Rep. 7, 1–16. 10.1038/s41598-017-14039-y 29066782 PMC5655687

[B52] Vinceti M. Filippini T. Del Giovane C. Dennert G. Zwahlen M. Brinkman M. (2018). Selenium for Preventing Cancer. Database Syst. Rev. 29, 6491296. 10.1002/14651858.CD005195.pub4 PMC6491296 29376219

[B53] Wang B. Feng W. Wang M. Wang T. Gu Y. Zhu M. (2008). Acute Toxicological Impact of Nano- and Submicro-Scaled Zinc Oxide Powder on Healthy Adult Mice. J. Nanopart Res. 10 (2), 263–276. 10.1007/s11051-007-9245-3

[B54] Wang W. Mai K. Zhang W. Xu W. Ai Q. Liufu Z. (2012). Dietary Selenium Requirement and its Toxicity in Juvenile Abalone *Haliotis Discus Hannai Ino* . Aquaculture 330-333, 42–46. 10.1016/j.aquaculture.2011.11.032

[B55] Wehmas L. C. Anders C. Chess J. Punnoose A. Pereira C. B. Greenwood J. A. (2015). Comparative Metal Oxide Nanoparticle Toxicity Using Embryonic Zebrafish. Toxicol. Rep. 2, 702–715. 10.1016/j.toxrep.2015.03.015 26029632 PMC4443491

[B56] Westerfield M. (2007). The Zebrafish Book, 5th Edition; A Guide for the Laboratory Use of Zebrafish (*Danio rerio*). Eugene: University of Oregon Press.

[B57] Wong S. W. Y. Leung P. T. Y. Djurišić A. B. Leung K. M. Y. (2010). Toxicities of Nano Zinc Oxide to Five marine Organisms: Influences of Aggregate Size and Ion Solubility. Anal. Bioanal. Chem. 396, 609–618. 10.1007/s00216-009-3249-z 19902187

[B58] Zemheri-Navruz F. Acar Ü. Yılmaz S. (2020). Dietary Supplementation of Olive Leaf Extract Enhances Growth Performance, Digestive Enzyme Activity and Growth Related Genes Expression in Common Carp *Cyprinus carpio* . Gen. Comp. Endocrinol. 296, 113541. 10.1016/j.ygcen.2020.113541 32585215

[B59] Zhao X. Wang S. Wu Y. You H. Lv L. (2013). Acute ZnO Nanoparticles Exposure Induces Developmental Toxicity, Oxidative Stress and DNA Damage in Embryo-Larval Zebrafish. Aquat. Toxicol. 136-137, 49–59. 10.1016/j.aquatox.2013.03.019 23643724

[B60] Zhou X. Wang Y. Gu Q. Li W. (2009). Effects of Different Dietary Selenium Sources (Selenium Nanoparticle and Selenomethionine) on Growth Performance, Muscle Composition and Glutathione Peroxidase Enzyme Activity of Crucian Carp (*Carassius auratus Gibelio*). Aquaculture 291, 78–81. 10.1016/j.aquaculture.2009.03.007

[B61] Zhu X. Wang J. Zhang X. Chang Y. Chen Y. (2009). The Impact of ZnO Nanoparticle Aggregates on the Embryonic Development of Zebrafish (*Danio rerio*). Nanotechnology 20 (19), 195103. 10.1088/0957-4484/20/19/195103 19420631

[B62] Zhu X. Zhu L. Duan Z. Qi R. Li Y. Lang Y. (2008). Comparative Toxicity of Several Metal Oxide Nanoparticle Aqueous Suspensions to Zebrafish (*Danio rerio*) Early Developmental Stage. J. Environ. Sci. Health A 43, 278–284. 10.1080/10934520701792779 18205059

