# Supplementary material for: Selenium and Zinc Oxide Multinutrient Supplementation Enhanced Growth Performance in Zebra Fish by Modulating Oxidative Stress and Growth-Related Gene Expression
Source: Front Bioeng Biotechnol. 2021 Oct 6;9:721717. doi: 10.3389/fbioe.2021.721717 (PMC8528278; doi:10.3389/fbioe.2021.721717)
Supplement: Supplementary file 1 [file DataSheet1.docx]

Supplementary information

Fasil Dawit Moges, Hamida Hamdi, Amal Al-Barty, Abeer Abu Zaid, S.K.S. Parashar, Biswadeep Das

Supplementary Figure S1. Hydrodynamic diameter (nm) distribution for SeNPs and ZnONPs (10 hr ball milling time) in aqueous solution for independent experiments. The hydrodynamic size distribution was 238 ± 14 nm and 252 ± 15 nm for SeNPs and ZnONPs, respectively.


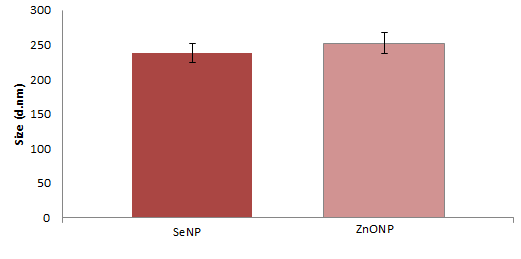


Supplementary Figure S2. *Percentage of Survivability for all four treatments across the experimental period.*

Supplementary Table S1: Primers for the growth related zebrafish genes used for the qRT-PCR

| **Primer name** | **Primer (5’-3’)** | **Amplicon size** | **Tm °C** |
| --- | --- | --- | --- |
| Growth hormone | GCATCAGCGTGCTCATCAAG | 110 | 58-62 |
|  | TGGTCTCCCCTACGGTCAG |  |  |
| Insulin like growth factor | AGTACCCACACCCTCTCACT | 132 |  |
|  | AAAGCCCCTGTCTCCACAC |  |  |
| Beta actin | CCTCTCTTGCTCCTTCCACC | 150 |  |
|  | TACTCCTGCTTGCTGATCCAC |  |  |
